# The association of a diabetes-specific health plan with ER and inpatient hospital utilization: a natural experiment for translation in diabetes (NEXT-D)

**DOI:** 10.1186/1748-5908-10-S1-A4

**Published:** 2015-08-14

**Authors:** Tannaz Moin, W Neil Steers, Susan L Ettner, O Kenrik Duru, Norman Turk, Romain Neugebauer, Charles Chan, Robert H Luchs, Sam Ho, Carol M Mangione

**Affiliations:** Department of Medicine, VA Greater Los Angeles Healthcare System, Los Angeles, CA 90073 USA; VA HSR&D Center for the Study of Healthcare Innovation, Implementation and Policy, Los Angeles, CA 91343 USA; David Geffen School of Medicine, University of California, Los Angeles, CA 90024 USA; Jonathan and Karin Fielding School of Public Health, University of California, Los Angeles, CA 90024 USA; Division of Research, Kaiser Permanente Northern California, Oakland, CA 94612 USA; UnitedHealthcare, Minnetonka, MN 55343 USA

## Context

The Diabetes Health Plan (DHP) is a disease-specific health plan for patients with diabetes and pre-diabetes which offers reduced cost sharing for pharmacy, office visits and disease management. The DHP was purchased by some employers and not by others, creating a unique opportunity to conduct a rigorous evaluation of a real-world, naturally occurring intervention, i.e., a natural experiment.

## Objectives

Examine the association of employer purchase of the DHP with emergency room (ER) and inpatient hospital utilization among employees and covered dependents with diabetes and pre-diabetes.

## Design

A quasi-experimental design with the employer as the unit of analysis, comparing changes in mean ER and hospital utilization over a 3-year period. We used inverse probability weighting (IPW) to adjust for differences between employers that purchased the DHP and employers that purchased standard plans. We report estimated differences as Average Treatment Effects on the Treated (ATET). Setting: Large, national private insurer that offers health plans to public and private employers. Participants: We aggregated eligibility and claims data from covered employees and dependents with diabetes and pre-diabetes (n = 74,058) to the employer level. The analysis included 9 employer groups that purchased the DHP in 2009 or 2010 (N = 7,004 employees and dependents) and 183 control employer groups that purchased standard plans in 2009 or 2010 (N = 67,054 employees and dependents). Main Outcome Measure: Mean rates of ER and inpatient hospital utilization.

## Results

DHP purchase was associated with 2.4 and 1.8 percentage-point reductions in adjusted mean rates of any ER utilization, representing 13% and 10% relative reductions at 1 and 2-years post-DHP (p = 0.012 and p = 0.046, respectively). There was no evidence of significant associations between DHP purchase and adjusted hospital utilization.

## Conclusion

Employer groups purchasing diabetes-specific health benefit designs such as the DHP may experience lower rates of resource-intensive services such as ER utilization.

## Sources of funding

This study was jointly funded by the Centers for Disease Control and Prevention (Division of Diabetes Translation) and the National Institute of Diabetes and Digestive and Kidney Diseases as part of the Natural Experiments for the Translation of Diabetes (NEXT-D) Study (Grant number DP002722).

